# Robust Multiple Servers Architecture Based Authentication Scheme Preserving Anonymity

**DOI:** 10.3390/s19143144

**Published:** 2019-07-17

**Authors:** Huawei Wang, Dianli Guo, Hua Zhang, Qiaoyan Wen

**Affiliations:** 1 State Key Laboratory of Networking and Switching Technology, Beijing University of Posts and Telecommunications, Beijing 100876, China; 2 The 6th Research Institute of China Electronics Corporation, Beijing 100083, China

**Keywords:** authentication, anonymity, BAN-logic, biometrics, multiple server

## Abstract

Recently, many dynamic ID based remote user authentication schemes using smart card have been proposed to improve the security in multiple servers architecture authentication systems. In 2017, Kumari and Om proposed an anonymous multi-server authenticated key agreement scheme, which is believed to be secure against a range of network attacks. Nevertheless, in this paper we reanalyze the security of their scheme, and show that the scheme is vulnerable to impersonation attack and server spoofing attack launched by any adversary without knowing any secret information of the victim users. In addition, their protocol fails to achieve the claimed user privacy protection. For handling these aforementioned shortcomings, we introduce a new biometric-based authentication scheme for multi-server architecture preserving user anonymity. Besides, Burrows—Abadi—Needham (BAN)-logic validated proof and discussion on possible attacks demonstrate the completeness and security of our scheme, respectively. Further, the comparisons in terms of security analysis and performance evaluation of several related protocols show that our proposal can provide stronger security without sacrificing efficiency.

## 1. Introduction

In the multiple servers architecture based authentication system, registration center, service providing servers and users are major participants. Registration center is the trusted party to administrate all the involved users and servers in the system. Servers provide network services and legitimate users can access these services. Compared with the conventional two-party authentication system, a multi-server architecture based authentication system offers registration procedure one time and allows users to access services from multiple servers. The latter obliterates the inappropriateness that users should perform stuffy reduplicative registration in each server.

In 2004, Das et al. [1] proposed a dynamic ID based remote user authentication scheme using smart card. Since then, many dynamic ID authentication schemes are published to enhance the security properties and reduce the communication and computation costs [2,3,4,5,6,7,8,9,10,11,12]. However, these schemes are designed for single-server architecture which are not suitable for a multi-server environment.

For fulfilling the particularity of the multi-server architecture, ample authentication schemes designed for the multi-server environment have been investigated by researchers. In 2009, Liao and Wang [13] proposed a remote user authentication scheme for multi-server architecture preserving user anonymity to eliminate the risk of ID-theft. Nevertheless, their scheme is proved to be susceptible to insider attack, masquerade attack and fail to provide a mutual authentication. Later on, Hsiang and Shih [14] introduced a remedied protocol to solve the above security flaws. Unfortunately, Sood et al. [15] reanalyzed their scheme and pointed out that it is vulnerable to replay attack, impersonation attack and stolen smart card attack. Meanwhile, they presented a novel multiple servers based authentication scheme. After that, many multi-server authentication schemes have been proposed to strengthen the security and improve the efficiency [16,17,18,19,20,21,22,23,24,25,26].

In 2014, Chuang and Chen [16] proposed an anonymous multi-server authentication key agreement scheme using smart cards, password and biometrics. However, Kumari and Om [23] identified the vulnerabilities of their scheme, such as being insecure against DoS attack, user/server impersonation attack, stolen smart card attack, and failing to achieve perfect forward secrecy. For obliterating the aforementioned shortcomings of Chuang et al.’s scheme, they proposed an enhanced protocol for a multiple servers authentication system, which offered non-repudiation utilizing RSA digital signature. Moreover, the authors stated that their proposal possessed all required security properties and resisted all the network attacks. Unluckily, in this paper, we reexamine the security of Kumari et al.’s RSA cryptosystem based authentication scheme and indicate that their scheme falls short to withstand impersonation attack and server spoofing attack. Specifically, any adversary could break through their scheme easily, even without the knowledge of the victim’s user information. Moreover, adversaries could create test scenarios to execute the brute force attack and reveal users’ low entropy identities. For the purpose of surmounting the identified vulnerabilities, we further devise an improved biometric-based multi-server authentication scheme with a distinctive policy compared with the original. Note that, Burrows—Abadi—Needham (BAN)-logic, one of the important formal methods focusing on evaluating the beliefs of participants in authentication system, is put forward to certify the validity of our proposal. Finally, the security and performance analysis are discussed to observe that the proposed protocol is superior to other related schemes.

This paper is organized as follows. We introduce the basic concept of fuzzy extraction in Section 2. Then, in Section 3 and Section 4 we briefly review Kumari et al.’s scheme and identify its security flaws, respectively. Next, we propose a new robust authentication scheme in Section 5 and analyze its security in Section 6. Subsequently, in Section 7 we compare the performance of our new protocol with the previous schemes. Finally, the paper is concluded in Section 8.

## 2. Preliminaries

In this section, we briefly introduce the basic concept of fuzzy extractor, for more details please refer to [27]. In 1999, Juels and Wattenberg fetched out the definition of fuzzy extractor which focused on verifying the legality of users by biometric template. Noticeably, it could deal with non-uniformity and error tolerance. Concretely, it could output a uniform key *R* with an auxiliary *P* and non-uniform noisy biometric input $B^{*}$ by employing reproducible extraction, which was an error tolerant approach. The auxiliary string *P* to recover authentication key *R* is a public parameter and does certainly not compromise secrecy of *R*. Probabilistic generation algorithm Gen and deterministic reproduction algorithm Rep are efficient procedures of fuzzy extractor with parameters (m,l,t,ϵ), which are detailed as follows.

- Gen: Inputs biometric template *B*, outputs an authenticated value $R\in {\left\{0,1\right\}}^{l}$ and an auxiliary value $P\in {\left\{0,1\right\}}^{*}$.
- Rep: For all $B,\;B^{\prime }$, if $dis(B,B^{\prime })\leq t$ and 〈R,P〉←Gen(B), then $Rep(B^{\prime },P)=R$.

We list the notations used throughout this paper in Table 1.

## 3. Review of Kumari and Om’s Scheme

**Figure 1:**
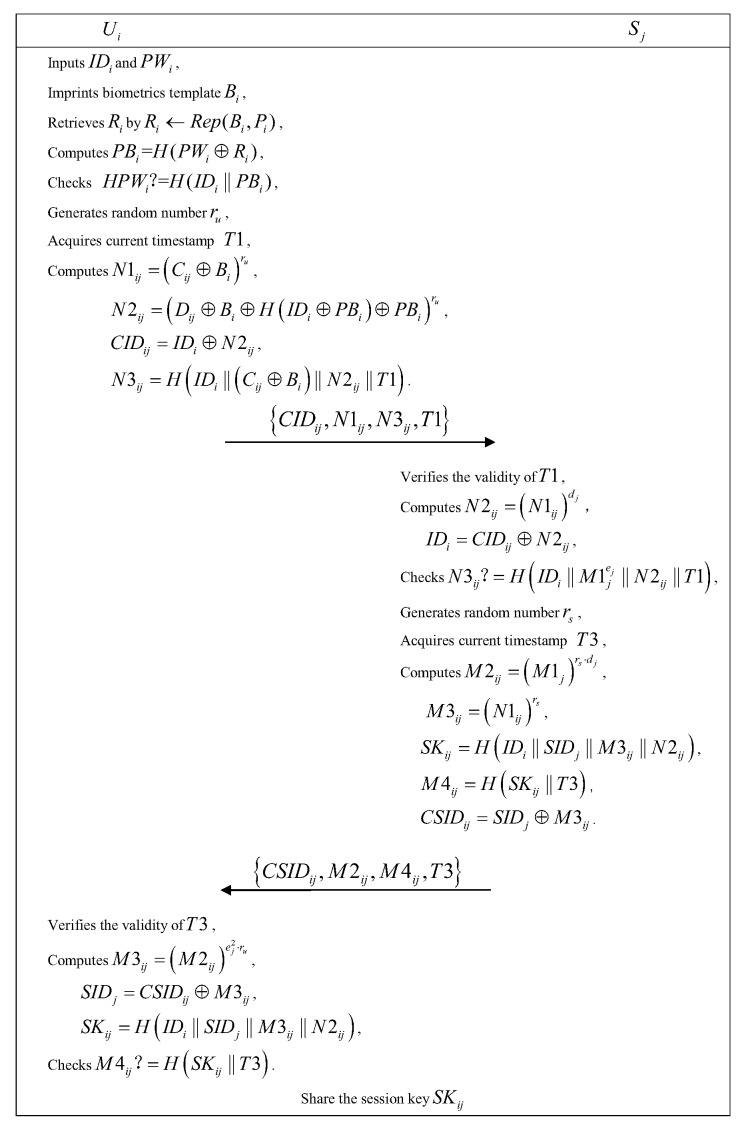
Login phase and authentication phase.

In this section, we briefly describe Kumari and Om’s [23] multi-server architecture based authentication scheme. It consists of initialization, registration, login, authentication and password changing phases. In Figure 1, we describe in detail the login and authentication phases in the form of infographics.

### 3.1. Initialization Phase

Registration center RC chooses a secret value $X_{c}$ and two distinct large prime numbers *p*, *q*. Subsequently, it calculates n=p×q and ϕ(n)=(p−1)×(q−1). $X_{c}$ is the master secret key and only kept by RC. p,q could be destroyed to avoid leaking.

### 3.2. Registration Phase

#### 3.2.1. Server Registration

Application server $S_{j}$ transmits its identification $SID_{j}$ to RC and applies for the jurisdiction to offer network services. RC selects a random number $e_{j}\in \left(1,\phi \left(n\right)\right)$ with $gcd(e_{j},\phi \left(n\right))=1$. Then it computes and seeks out $d_{i}$ such that $e_{j}\times d_{j}\equiv 1mod\left(\phi \left(n\right)\right)$. Finally, RC sends the credentials $\left\{M1_{j},e_{j},d_{j},n\right\}$ to $S_{j}$ via a secured communication channel, where $M1_{j}=H\left(SID_{j}∥X_{c}\right)$, $d_{j}$ are kept secret and $e_{j}$, *n* are announced as public values.

#### 3.2.2. User Registration

Step 1: $U_{i}$ firstly imprints his/her biometrics and uses fuzzy extractor to obtain authenticated value $R_{i}$ and auxiliary value $P_{i}$ such that $Gen\left(B_{i}\right)=\left(R_{i},P_{i}\right)$. Then, he/she selects identity $ID_{i}$ and password $PW_{i}$ to calculate $PB_{i}=H\left(PW_{i}⊕R_{i}\right)$ and sends $\left\{ID_{i},PB_{i},P_{i}\right\}$ to registration center for registration.

Step 2: Upon receiving registration request from $U_{i}$, RC computes $HPW_{i}=H\left(ID_{i}∥PB_{i}\right)$, $A_{i}=H\left(ID_{i}∥X_{c}\right)⊕PB_{i}$, $B_{i}=H\left(ID_{i}⊕PB_{i}\right)⊕A_{i}$, $C_{ij}=B_{i}⊕M1_{j}^{e_{j}}$ and $D_{ij}=M1_{j}⊕H\left(ID_{i}∥X_{c}\right)$. Then, RC transmits the smart card contained $\left\{B_{i},C_{ij},D_{ij},HPW_{i},P_{i},H\left(\cdot \right)\right\}$ to $U_{i}$ securely.

### 3.3. Login Phase

$U_{i}$ inserts his/her smart card into the terminal and inputs identity $ID_{i}$, password and the biometric template $B_{i}$ imprinted at the sensor. The smart card will execute the following procedure.

Step 1: Performs reproduction algorithm $R_{i}^{*}=Rep\left(B_{i},P_{i}\right)$ and computes $PB_{i}^{*}=H\left(PW_{i}⊕R_{i}^{*}\right)$, $HPW_{i}^{*}=H\left(ID_{i}∥PB_{i}^{*}\right)$.

Step 2: Verifies the equivalence of $HPW_{i}^{*}$ and the stored value $HPW_{i}$. If they are equal, proceeds to next steps; otherwise, terminates this session immediately.

Step 3: Generates a random number $r_{u}$ and acquires the current timestamp T1 to calculate $N1_{ij}={\left(C_{ij}⊕B_{i}\right)}^{r_{u}}$, $N2_{ij}={\left(D_{ij}⊕B_{i}⊕H\left(ID_{i}⊕PB_{i}\right)⊕PB_{i}\right)}^{r_{u}}$, $CID_{ij}=ID_{i}⊕N2_{ij}$, $N3_{ij}=H(ID_{i}∥\left(C_{ij}⊕B_{i}\right)∥N2_{ij}∥T1)$.

Step 4: Submits the login request message $\left\{CID_{ij},N1_{ij},N3_{ij},T1\right\}$ to $S_{j}$.

### 3.4. Authentication Phase

On receiving the login request $\left\{CID_{ij},N1_{ij},N3_{ij},T1\right\}$ from $U_{i}$ at T2, $S_{j}$ verifies the validity of T1 by checking T2−T1 whether less or equal than the permissible time interval △T for a transmission delay. If so, continues to perform the following steps; else, $S_{j}$ aborts the login session.

Step 1: $S_{j}$ computes $N2_{ij}={\left(N1_{ij}\right)}^{d_{j}}$, $ID_{i}=CID_{ij}⊕N2_{ij}$, $N3_{ij}=H(ID_{i}∥M1_{j}^{e_{j}}∥N2_{ij}∥T1)$ with the known credential $d_{j}$.

Step 2: Then $S_{j}$ verifies the computed $N3_{ij}$ with the received one. If the equation does not hold, terminates the session; on the contrary, continues to execute the further steps.

Step 3: $S_{j}$ acquires the current timestamp T3 and generates a random number $r_{s}$ to compute $M2_{ij}={\left(M1_{j}\right)}^{r_{s}\cdot d_{j}}$, $M3_{ij}={\left(N1_{ij}\right)}^{r_{s}}$, $SK_{ij}=H(ID_{i}∥SID_{j}∥M3_{ij}∥N2_{ij})$, $M4_{ij}=H\left(SK_{ij}∥T3\right)$, $CSID_{ij}=SID_{j}⊕M3_{ij}$.

Step 4: Subsequently, $S_{j}$ responses to $U_{i}$ the replied mutual authentication message $\left\{CSID_{ij},M2_{ij},M4_{ij},T3\right\}$.

Step 5: Upon receiving the response message from $S_{j}$ at T4, $U_{i}$ checks whether T4−T3≤△T. If it does not hold, $U_{i}$ gives up this login procedure; otherwise, the smart card computes $M3_{ij}={\left(M2_{ij}\right)}^{e_{j}^{2}\cdot r_{u}}$, $SID_{j}=CSID_{ij}⊕M3_{ij}$, $SK_{ij}=H(ID_{i}∥SID_{j}∥M3_{ij}∥N2_{ij})$, $M4_{ij}=H\left(SK_{ij}∥T3\right)$.

Step 6: Afterwards, the smart card checks the equivalence of the computed $M4_{ij}$ and the received one. If they are not equal, the authentication fails; else, $U_{i}$ confirms $S_{j}$ is authentic and the mutual authentication is completed. Finally, $U_{i}$ and $S_{j}$ share a current session key $SK_{ij}$.

### 3.5. Password Changing Phase

In the procedure of password changing phase, $U_{i}$ could update her/his password offline. Firstly, he/she should insert smart card into the device and input $ID_{i}$, $PW_{i}$, the biometric template $B_{i}$. Then, the smart card verifies the legitimation of $U_{i}$ to launch the following steps.

Step 1: Computes $R_{i}=Rep\left(B_{i},P_{i}\right)$, $PB_{i}=H\left(PW_{i}⊕R_{i}\right)$ and compares the stored value $HPW_{i}$ equals to the computed $H(ID_{i}∥PB_{i})$. If they are not equal, the smart card terminates this session; otherwise, the smart card continues to compute $A_{i}=B_{i}⊕H\left(ID_{i}⊕PB_{i}\right)$, $H\left(ID_{i}∥X_{c}\right)=A_{i}⊕PB_{i}$, $M1_{j}^{e_{j}}=C_{ij}⊕B_{i}$.

Step 2: Subsequently, $U_{i}$ is allowed to input a new password $PW_{i}^{new}$. The smart card calculates $PB_{i}^{new}=H\left(PW_{i}^{new}⊕R_{i}\right)$, $HPW_{i}^{new}=H\left(ID_{i}∥PB_{i}^{new}\right)$, $A_{i}^{new}=H\left(ID_{i}∥X_{c}\right)⊕PB_{i}^{new}$, $B_{i}^{new}=H\left(ID_{i}⊕PB_{i}^{new}\right)⊕A_{i}^{new}$, $C_{ij}^{new}=B_{i}^{new}⊕M1_{j}^{e_{j}}$.

Step 3: Finally, the smart card replaces $\left\{B_{i},C_{ij},HPW_{i}\right\}$ with the new parameters to finish the password change phase.

## 4. Cryptanalysis of Kumari and Om’s Scheme

In this phase, we show that Kumari and Om’s protocol is vulnerable to impersonation attack, server spoofing attack and fails to protect user anonymity. Their scheme is thoroughly broken down by any malicious user in the multi-server authentication system, even when one knows nothing about the victim user. The detailed demonstration is described as follows.

### 4.1. Impersonation Attacks

Consider a legitimate but malicious user $U_{A}$ in the multiple servers authentication system, he/she can obtain $M1_{j}^{e_{j}}=C_{Aj}⊕B_{A}$ and $M1_{j}=D_{Aj}⊕B_{A}⊕H\left(ID_{A}⊕PB_{A}\right)⊕PB_{A}$ with secret parameters stored in his/her own smart card. Then, the adversary can further impersonate any legal user (even a non-existent user) to unauthorized access $S_{j}$.

In the login phase, $U_{A}$ randomly selects a string $ID_{k}$ with the format of identity and computes $N1_{kj}={\left(M1_{j}^{e_{j}}\right)}^{r_{A}}=M1_{j}^{e_{j}\cdot r_{A}}$, $N2_{kj}={\left(M1_{j}\right)}^{r_{A}}$, $CID_{kj}=ID_{k}⊕N2_{kj}$, $N3_{kj}=H(ID_{k}∥M1_{j}^{e_{j}}∥N2_{kj}∥T1_{k})$, where $r_{A}$ and $T1_{k}$ are generated random number and acquired current timestamp respectively. Subsequently, he/she transmits the forged login request $\left\{CID_{kj},N1_{kj},N3_{kj},T1_{k}\right\}$ to $S_{j}$.

After receiving the forged login request, $S_{j}$ checks the validity of $T1_{k}$ and calculates $N2_{kj}={\left(N1_{kj}\right)}^{d_{j}}$, $ID_{k}=CID_{kj}⊕N2_{kj}$, $N3_{kj}=H(ID_{k}∥M1_{j}^{e_{j}}∥N2_{kj}∥T1_{k})$ with the known credential $d_{j}$. Obviously, the computed $N3_{kj}$ is consistent to the forged one in the login request, that is, the verification of $U_{A}$ is successful and $S_{j}$ accepts the login request of the adversary. Subsequently, $S_{j}$ computes $M2_{kj}={\left(M1_{j}\right)}^{r_{s}\cdot d_{j}}$, $M3_{kj}={\left(N1_{kj}\right)}^{r_{s}}$, $SK_{kj}=H(ID_{k}∥SID_{j}∥M3_{kj}∥N2_{kj})$, $M4_{kj}=H\left(SK_{kj}∥T3\right)$, $CSID_{kj}=SID_{j}⊕M3_{kj}$ with the random number $r_{s}$ and timestamp T3, and replies $\left\{CSID_{kj},M2_{kj},M4_{kj},T3\right\}$ to the adversary.

After that, the adversary computes $M3_{kj}={\left(M2_{kj}\right)}^{e_{j}^{2}\cdot r_{A}}$, $SID_{j}=CSID_{kj}⊕M3_{kj}$, $SK_{kj}=H(ID_{k}∥SID_{j}∥M3_{kj}∥N2_{kj})$. Finally, $U_{A}$ obtains the session key $SK_{kj}$ and uses it to communicate with $S_{j}$. Hence, the adversary successfully accesses the service providing server unauthorized.

### 4.2. Failure of Preserving Anonymity

As described above, a legitimate but malicious user $U_{A}$ could obtain $M1_{j}^{e_{j}}$ with his/her own secret values. Suppose that $U_{A}$ intercepts $U_{i}$’s login request $\left\{CID_{ij},N1_{ij},N3_{ij},T1\right\}$ in a prior transaction, he/she could easily get $U_{i}$’s identity by the brute force attack. In the following we present the concrete procedures.

Step 1. Firstly, let $ID_{i}^{*}$ be an identity candidate in the identity space. Subsequently, the adversary computes $N2_{ij}^{*}=CID_{ij}⊕ID_{i}^{*}$.

Step 2. Secondly, the adversary checks $N3_{ij}?=H(ID_{i}^{*}∥M1_{j}^{e_{j}}∥N2_{ij}^{*}∥T1)$ to verify the correctness of chosen candidate $ID_{i}^{*}$.

Step 3. The adversary performs Steps 1 and 2 repeated with another candidate in the identity space until the correct $ID_{i}$ such that $N3_{ij}=H(ID_{i}∥M1_{j}^{e_{j}}∥\left(CID_{ij}⊕ID_{i}\right)∥T1)$ is found.

Actually, the above attack could be executed effectively since the amount of identity space is limited. The primary causes of this problem are the inherently restricted human cognition and the limitation of identity format.

### 4.3. Server Spoofing Attack

In Kumari and Om’s protocol, a legitimate but malicious user $U_{A}$ also can masquerade as an authorized server. Based on the description in the above analysis, $U_{A}$ can obtain $U_{i}$’s identity $ID_{i}$ by employing feasible brute force attack and record the identity $SID_{j}$ of $S_{j}$ in a prior session. Furthermore, he/she also needs to intercept the mutual authentication message $\left\{CSID_{ij},M2_{ij},M4_{ij},T3\right\}$ replied to $U_{i}$ from $S_{j}$ in a previous session, and records a pair values $\left(M2_{ij},M3_{ij}\right)=\left(M2_{ij},CSID_{ij}⊕SID_{j}\right)$. Noticeably, the adversary merely does preparatory work one time, rather than repetitively recording these values before performing each server spoofing attack. The concrete description of server spoofing attack is shown as follows.

Step 1: Suppose that $U_{i}$ requests to access $S_{j}$ with $\left\{CID_{ij},N1_{ij},N3_{ij},T1\right\}$. The adversary selects a random number $r_{A}$ and computes

$$M2_{ij}^{*}={\left(M2_{ij}\right)}^{r_{A}}={\left(M1_{j}\right)}^{r_{s}\cdot r_{A}\cdot d_{j}},$$

$$M3_{ij}^{*}={\left(M3_{ij}\right)}^{r_{A}}={\left(N1_{ij}\right)}^{r_{s}\cdot r_{A}},$$

$$SK_{ij}^{*}=H(ID_{i}∥SID_{j}∥M3_{ij}^{*}∥\left(CID_{ij}⊕ID_{i}\right)),$$

$$M4_{ij}^{*}=H\left(SK_{ij}^{*}∥T3_{A}\right),$$

$$CSID_{ij}^{*}=SID_{j}⊕M3_{ij}^{*}$$

with the values previously recorded. Afterwards, sends the forged response $\left\{CSID_{ij}^{*},M2_{ij}^{*},M4_{ij}^{*},T3_{A}\right\}$ to $U_{i}$.

Step 2: After receiving the forged reply, $U_{i}$ computes

$$M3_{ij}^{*}={\left(M2_{ij}^{*}\right)}^{e_{j}^{2}\cdot r_{u}}={\left(M1_{j}\right)}^{e_{j}\cdot r_{u}\cdot r_{s}\cdot r_{A}}={\left(N1_{ij}\right)}^{r_{s}\cdot r_{A}},$$

$$SID_{j}=CSID_{ij}^{*}⊕M3_{ij}^{*},$$

$$SK_{ij}^{*}=H(ID_{i}∥SID_{j}∥M3_{ij}^{*}∥N2_{ij}).$$

Obviously, the computed $H(SK_{ij}^{*}∥T3_{A})$ is equal to the received $M4_{ij}^{*}$. Hence, $U_{i}$ authenticates the adversary successfully and communicates with him/her. The major contributor of the network flaw is the allelomorphism of array $\left(M2_{ij},M3_{ij}\right)$—any attacker could reconstitute these two values by performing an exponentiation with exponent $r_{A}$ respectively.

## 5. Our Scheme

**Figure 2:**
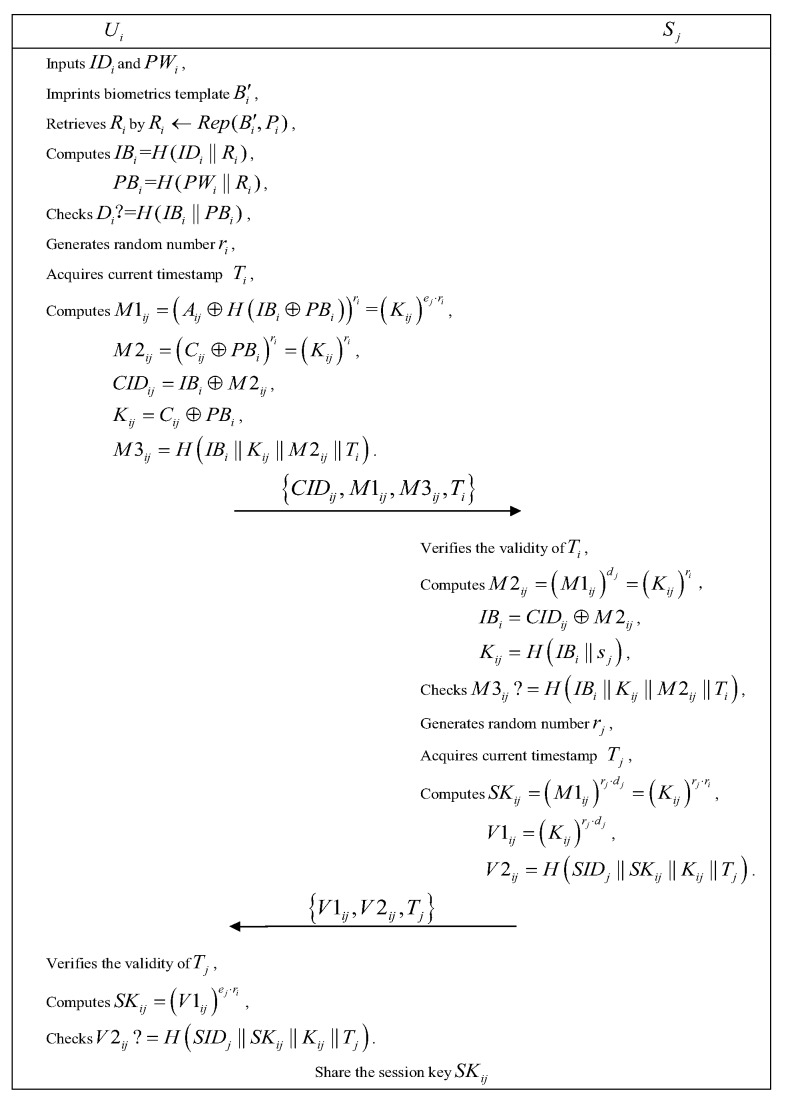
Login phase and authentication phase.

Herein, we propose a novel multiple servers architecture based authentication scheme with biometrics, which contains five phases, namely initialization phase, registration phase, login phase, authentication phase and password changing phase. Furthermore, we depict the login and authentication phases in Figure 2.

### 5.1. Initialization Phase

Registration center RC initializes the authentication system with secret value $X_{c}$ and two distinct large primes *p*, *q*. Then, it keeps $\left\{X_{c}\right\}$ secret and publishes the public parameters {n,ϕ(n)}, where n=p×q, ϕ(n)=(p−1)×(q−1). Finally, RC obliterates the two values p,q.

### 5.2. Registration Phase

This segment contains two sub-phases: server registration and user registration. Service providing servers and users apply for authorization of registration center through the following procedures, respectively.

#### 5.2.1. Server Registration

Similar to the original protocol, service providing server $S_{j}$ sends its identity $SID_{j}$ to RC for registration. After receiving the registration request, it seeks out two large numbers $e_{j}\in \left(1,\phi \left(n\right)\right)$ and $d_{j}$ such that $gcd(e_{j},\phi \left(n\right))=1$ and $e_{j}\times d_{j}\equiv 1mod\left(\phi \left(n\right)\right)$, computes $s_{j}=H\left(SID_{j}∥X_{c}\right)$. Afterwards, RC transmits the calculated credentials $\left\{s_{j},e_{j},d_{j},n\right\}$ to $S_{j}$. $S_{j}$ publishes $\left\{e_{j},n\right\}$ and keeps $\left\{s_{j},d_{j}\right\}$ as secret keys.

#### 5.2.2. User Registration

Step 1: $U_{i}$ imprints the biometrics $B_{i}$ and invokes the fuzzy extractor to generate $\left(R_{i},P_{i}\right)\leftarrow Gen\left(B_{i}\right)$. Subsequently, he/she calculates $IB_{i}=H\left(ID_{i}∥R_{i}\right)$ and $PB_{i}=H\left(PW_{i}∥R_{i}\right)$ with the selected identity and password. After that, $U_{i}$ registers in RC with $\left\{IB_{i},PB_{i}\right\}$.

Step 2: Then, RC computes $K_{ij}=H\left(IB_{i}∥s_{j}\right)$ with each service providing server secret key $\left\{s_{1},s_{2},\cdot \cdot \cdot ,s_{k}\right\}$. RC continues to calculate $A_{ij}={\left(K_{ij}\right)}^{e_{j}}⊕H\left(IB_{i}⊕PB_{i}\right)$, $C_{ij}=K_{ij}⊕PB_{i}$ and $D_{i}=H\left(IB_{i}∥PB_{i}\right)$. Afterwards, RC personalizes the smart card with the $\left\{\left(A_{i1},A_{i2},\cdot \cdot \cdot ,A_{ik}\right),\left(C_{i1},C_{i2},\cdot \cdot \cdot ,C_{ik}\right),D_{i}\right\}$ and sends it to $U_{i}$ via a secure channel.

### 5.3. Login Phase

Step 1: $U_{i}$ inserts his/her smart card into the card reader and inputs $ID_{i}$, $PW_{i}$, the imprinted biometric template $B_{i}^{\prime }$. Then the smart card recovers the value $R_{i}$ through $R_{i}\leftarrow Rep\left(B_{i}^{\prime },P_{i}\right)$, computes $IB_{i}=H\left(ID_{i}∥R_{i}\right)$, $PB_{i}=H\left(PW_{i}∥R_{i}\right)$, and verifies whether the computed $H(IB_{i}∥PB_{i})$ equals to the stored $D_{i}$ or not. If they are consistent, continues to execute Step 2; otherwise, the login phase is aborted directly.

Step 2: The smart card generates a random number $r_{i}$ and calculates $M1_{ij}={\left(A_{ij}⊕H\left(IB_{i}⊕PB_{i}\right)\right)}^{r_{i}}={\left(K_{ij}\right)}^{e_{j}\cdot r_{i}}$, $M2_{ij}={\left(C_{ij}⊕PB_{i}\right)}^{r_{i}}={\left(K_{ij}\right)}^{r_{i}}$, $CID_{ij}=IB_{i}⊕M2_{ij}$, $K_{ij}=C_{ij}⊕PB_{i}$, $M3_{ij}=H(IB_{i}∥K_{ij}∥M2_{ij}∥T_{i})$, where $T_{i}$ is the acquired current timestamp.

Step 3: $U_{i}$ accesses $S_{j}$ with the login request $\left\{CID_{ij},M1_{ij},M3_{ij},T_{i}\right\}$.

### 5.4. Authentication Phase

Step 1: Upon receiving $U_{i}$’s login request at $T1_{i}$, $S_{j}$ checks the validity of $T_{i}$. If $T1_{i}-T_{i}\geq T$, $S_{j}$ rejects $U_{i}$’s login request; otherwise, it computes $M2_{ij}={\left(M1_{ij}\right)}^{d_{j}}={\left(K_{ij}\right)}^{r_{i}}$. Further, $S_{j}$ uses $M2_{ij}$ to recover $IB_{i}$ with $CID_{ij}⊕M2_{ij}$. Subsequently, it verifies the uniformity of the computed value $H(IB_{i}∥H\left(IB_{i}∥s_{j}\right)∥M2_{ij}∥T_{i})$ and the received $M3_{ij}$. If they are equal, the legitimacy of $U_{i}$ is ensured; on the contrary, $S_{j}$ discards the session immediately.

Step 2: After that, $S_{j}$ acquires the current timestamp $T_{j}$ and generates a random integer number $r_{j}$ to compute $SK_{ij}={\left(M1_{ij}\right)}^{r_{j}\cdot d_{j}}={\left(K_{ij}\right)}^{r_{j}\cdot r_{i}}$, $V1_{ij}={\left(K_{ij}\right)}^{r_{j}\cdot d_{j}}$, $V2_{ij}=H(SID_{j}∥SK_{ij}∥K_{ij}∥T_{j})$. Subsequently, it sends the response authentication message $\left\{V1_{ij},V2_{ij},T_{j}\right\}$ to $U_{i}$.

Step 3: Upon receiving the replied message at $T1_{j}$, the smart card verifies the validity of $T_{j}$. If $T1_{j}-T_{j}$ is less than or equal to the permissible time interval △T for a transmission delay, the authentication fails. Otherwise, the smart card calculates $SK_{ij}={\left(V1_{ij}\right)}^{e_{j}\cdot r_{i}}$ and $V2_{ij}^{*}=H(SID_{j}∥SK_{ij}∥K_{ij}∥T_{j})$. If $V2_{ij}^{*}=V2_{ij}$, $U_{i}$ confirms that $S_{j}$ is authentic and mutual authentication is completed successfully; on the contrary, the session will be terminated.

After finishing the above mutual authentication procedures, $S_{j}$ and $U_{i}$ agree on the session key $SK_{ij}$ for the future secure communication.

### 5.5. Password Changing Phase

These procedures are invoked whenever $U_{i}$ changes the overdue password with a new one.

Step 1: Similar to Step 1 of login phase, the smart card verifies the legitimacy of the card holder. If it confirms the validity of $U_{i}$, the smart card proceeds to Step 2; otherwise, it rejects the request of changing password.

Step 2: The smart card permits $U_{i}$ to enter a new password $PW_{i}^{new}$ to replace the original. Specifically, $U_{i}$ should enter the new one twice to prevent him/her from typing errors. Suppose that the entered passwords are unequal—the smart card requests $U_{i}$ to enter a new one two more times.

Step 3: After that, the smart card computes $PB_{i}^{new}=H\left(PW_{i}^{new}∥R_{i}\right)$, $A_{ij}^{new}=A_{ij}⊕H\left(IB_{i}⊕PB_{i}^{new}\right)$, $C_{ij}^{new}=C_{ij}⊕PB_{i}^{new}$, $D_{i}^{new}=H\left(IB_{i}∥PB_{i}^{new}\right)$. Then it replaces the original values with $\left\{\left(A_{i1}^{new},A_{i2}^{new}\cdot \cdot \cdot ,A_{ik}^{new}\right),\left(C_{i1}^{new},C_{i2}^{new},\cdot \cdot \cdot ,C_{ik}^{new}\right),D_{i}^{new}\right\}$.

## 6. Security Analysis and Discussion

### 6.1. Authentication Proof Based on BAN-Logic

Herein, we present the demonstration for the completeness of the proposed scheme through BAN-logic [28]. BAN-logic is one of the widely employed formal proofs for analyzing the trustworthiness of involved participants in authentication protocol.

In the following, we define some notations for the further BAN-logic analysis.

- P∣≡X: The principal P believes a statement *X* or P would be entitled to believe *X*.
- ♯(X): The formula *X* is fresh.
- P⇒X: The principal P has jurisdiction over the statement *X*.
- P◃X: The principal P sees the statement *X*.
- P∣∼X: The principal P once said the statement *X*.
- (X,Y): The formula *X* or *Y* is one part of the formula (X,Y).
- ${\langle X\rangle }_{Y}$: The formula *X* is combined with the formula *Y*.
- $P\overset{K}{⟷}Q$: The principals P and Q use the shared key *K* to communicate. Here, *K* will never be discovered by any principal except for P and Q.
- $P\overset{K}{⇌}Q$: *K* is shared secret known to P, Q, and possibly to one trusted by them.
- $SK_{ij}$: The session key used in the current session.

We present several logical postulates of BAN-logic as follows.

- The message-meaning rule: $\frac{P|\equiv Q\overset{K}{⇌}P,P◃{\langle X\rangle }_{K}}{P|\equiv Q|∼X}$.
- The freshness-conjuncatenation rule: $\frac{P|\equiv ♯(X)}{P|\equiv ♯(X,Y)}$.
- The nonce-verification rule: $\frac{P|\equiv ♯(X),P|\equiv Q|∼X}{P|\equiv Q|\equiv X}$.
- The jurisdiction rule: $\frac{P|\equiv Q\Rightarrow X,P|\equiv Q|\equiv X}{P|\equiv X}$, $\frac{P|\equiv (X,Y)}{P|\equiv X}$, $\frac{P◃(X,Y)}{P◃X}$, $\frac{P|\equiv Q|∼(X,Y)}{P|\equiv Q|∼X}$.

In the following, we present the verification goals based on the analytic procedures of BAN-logic.

- **Goal 1**: $U_{i}|\equiv \left(U_{i}\overset{SK_{ij}}{⟷}S_{j}\right)$
- **Goal 2**: $S_{j}|\equiv \left(U_{i}\overset{SK_{ij}}{⟷}S_{j}\right)$

Next, we present the idealized form of the proposed scheme which was arranged from generic type.

- Message 1: $U_{i}\to S_{j}$: $\left(CID_{ij},M1_{ij},{\langle IB_{i},M2_{ij},T_{i}\rangle }_{K_{ij}},T_{i}\right)$
- Message 2: $S_{j}\to U_{i}$: $\left(V1_{ij},{\langle SID_{j},SK_{ij},T_{j}\rangle }_{K_{ij}},T_{j}\right)$

In the following, we present some assumptions about the initial state of our proposed scheme to further analyze it.

- A.1: $U_{i}|\equiv \left(U_{i}\overset{K_{ij}}{⇌}S_{j}\right)$
- A.2: $S_{j}|\equiv \left(U_{i}\overset{K_{ij}}{⇌}U_{i}\right)$
- A.3: $U_{i}|\equiv ♯\left(T_{j}\right)$
- A.4: $S_{j}|\equiv ♯\left(T_{i}\right)$
- A.5: $S_{j}|\equiv U_{i}\Rightarrow \left(IB_{i},M2_{ij},T_{i}\right)$
- A.6: $U_{i}|\equiv S_{j}\Rightarrow \left(SID_{j},SK_{ij},T_{j}\right)$
- A.7: $S_{j}|\equiv r_{j}$

Next, we analyze the idealized form of the proposed protocol based on the aforementioned assumptions and logical postulates—the primary proof steps of BAN-logic are described in the following:

According to Message 1, we could prove:

$S_{j}◃\left(CID_{ij},M1_{ij},{\langle IB_{i},M2_{ij},T_{i}\rangle }_{K_{ij}},T_{i}\right)$.

According to the jurisdiction rule, we could prove:

$S_{j}◃{\langle IB_{i},M2_{ij},T_{i}\rangle }_{K_{ij}}$.

According to assumption A.2 and the message-meaning rule, we could prove:

$S_{j}|\equiv U_{i}|∼\left(IB_{i},M2_{ij},T_{i}\right)$.

According to the assumption A.4 and the freshness-conjuncatenation rule, we could prove:

$S_{j}|\equiv ♯\left(IB_{i},M2_{ij},T_{i}\right)$.

According to $S_{j}|\equiv U_{i}|∼\left(IB_{i},M2_{ij},T_{i}\right)$ and the nonce-verification rule, we could prove:

$S_{j}|\equiv U_{i}|\equiv \left(IB_{i},M2_{ij},T_{i}\right)$.

According to the assumption A.5 and the jurisdiction rule, we could prove:

$S_{j}|\equiv \left(IB_{i},M2_{ij},T_{i}\right)$.

According to the jurisdiction rule, we could prove:

$S_{j}|\equiv M2_{ij}$.

According to $SK_{ij}={\left(M2_{ij}\right)}^{r_{j}}$ and the assumption A.7, we could prove:

$S_{j}|\equiv \left(U_{i}\overset{SK_{ij}}{⟷}S_{j}\right)$ (**Goal 2**).

According to Message 2, we could prove:

$U_{i}◃\left(V1_{ij},{\langle SID_{j},SK_{ij},T_{j}\rangle }_{K_{ij}},T_{j}\right)$.

According to the jurisdiction rule, we could prove:

$U_{i}◃{\langle SID_{j},SK_{ij},T_{j}\rangle }_{K_{ij}}$.

According to assumption A.1 and the message-meaning rule, we could prove:

$U_{i}|\equiv S_{j}|∼\left(SID_{j},SK_{ij},T_{j}\right)$.

According to the assumption A.3 and the freshness-conjuncatenation rule, we could prove:

$U_{i}|\equiv ♯\left(SID_{j},SK_{ij},T_{j}\right)$.

According to $U_{i}|\equiv S_{j}|∼\left(SID_{j},SK_{ij},T_{j}\right)$ and the nonce-verification rule, we could prove:

$U_{i}|\equiv S_{j}|\equiv \left(SID_{j},SK_{ij},T_{j}\right)$.

According to the assumption A.6 and the jurisdiction rule, we could prove:

$U_{i}|\equiv \left(SID_{j},SK_{ij},T_{j}\right)$.

According to the jurisdiction rule, we could prove:

$U_{i}|\equiv SK_{ij}$,

$U_{i}|\equiv SID_{j}$.

According to $U_{i}|\equiv SK_{ij}$ and $U_{i}|\equiv SID_{j}$, we could prove:

$U_{i}|\equiv \left(U_{i}\overset{SK_{ij}}{⟷}S_{j}\right)$ (**Goal 1**).

### 6.2. Discussion on Possible Attacks

In this section, we present the security analysis in regard to a series of venomous network attacks and security properties to evaluate the proposed scheme.

#### 6.2.1. Preserve User Privacy

In the proposed scheme, $S_{j}$ can retrieve the identity information $IB_{i}=H\left(ID_{i}∥R_{i}\right)$ from the value $CID_{ij}=IB_{i}⊕M2_{ij}$ in the login request, which integrates with exponent $r_{i}$ of $K_{ij}$. $S_{j}$ keeps the secret key $d_{j}$ and can recover $M2_{ij}$ with another value $M1_{ij}$ in login request by computing $M2_{ij}=M1_{ij}^{d_{j}}$. In this way, the adversary either compromises $S_{j}$’s master secret key $d_{j}$ or solves the big integer factorization problem. Whereas, it is infeasible for him/her to obtain $IB_{i}$ in the above introduced method. Additionally, in our scheme, the dynamic identity $CID_{ij}$ is invoked by a hash value $IB_{i}$ of $U_{i}$’s identity and biometric template, rather than a low entropy identity $ID_{i}$. Hence, the adversary could not reveal user’s $ID_{i}$ by the attack introduced for breaching Kumari and Om’ protocol. Accordingly, our proposal is secure to against ID-theft attack and achieves user privacy protection.

#### 6.2.2. Off-Line Password Guessing Attack

The adversary could perform brute force attack to compromise the low entropy password with the eavesdropped session messages and revealed parameters stored in the smart card of victim users [29,30]. Because of this vulnerability, we introduce another security factor biometrics in our proposal. Concretely, $IB_{i}$, $PB_{i}$ are both attached with the secret value $R_{i}$ retrieved by legitimate biometric template $B_{i}$. Assume that the attacker has revealed the credential $D_{i}=H\left(IB_{i}∥PB_{i}\right)$ stored in the smart card, he/she has to guess identity $ID_{i}$, password $PW_{i}$ and secret value $R_{i}$ simultaneously. Notably, the length of secret value $R_{i}$ meets the requirements of information security. Thus, off-line password guessing attack is fruitless for our proposed scheme.

#### 6.2.3. Impersonation Attack

Impersonation attack means that an adversary forges a login request to masquerade legitimate users for unauthorized access to network services. It is indispensable for the adversary to generate an authenticated login request message $\left\{CID_{ij},M1_{ij},M3_{ij},T_{i}\right\}$, where $M1_{ij}={\left(A_{ij}⊕H\left(IB_{i}⊕PB_{i}\right)\right)}^{r_{i}}={\left(K_{ij}\right)}^{e_{j}\cdot r_{i}}$, $M2_{ij}={\left(C_{ij}⊕PB_{i}\right)}^{r_{i}}={\left(K_{ij}\right)}^{r_{i}}$, $CID_{ij}=IB_{i}⊕M2_{ij}$, $K_{ij}=C_{ij}⊕PB_{i}$, $M3_{ij}=H(IB_{i}∥K_{ij}∥M2_{ij}∥T_{i})$. From computational procedure of these parameters, we can obviously see that $K_{ij}$ and $IB_{i}$ are the key values to form them. In our proposal, $K_{ij}=H\left(IB_{i}∥s_{j}\right)$ is a unique secret value contributed by secrets of server and user, instead of a static value $M1_{j}$ of $S_{j}$ for each user in Kumari and Om’ protocol. The measure guarantees that users cannot abuse a unitary element to access servers illegally. On the other hand, the adversary also has no ability to calculate the verified login request without knowing $K_{ij}$. Consequently, the impersonation attack is trivial in our scheme.

#### 6.2.4. Server Spoofing Attack

Server spoofing attack indicates that someone (it could even be a legal but malicious server) pretends to be another server to deceive users. In order to perform the attack, the adversary should reply to $U_{i}$ a rightful authentication message $\left\{V1_{ij},V2ij,T_{j}\right\}$ likewise with the victim server, where $V1_{ij}={\left(K_{ij}\right)}^{r_{j}\cdot d_{j}}$, $V2_{ij}=H(SID_{j}∥SK_{ij}∥K_{ij}∥T_{j})$, $SK_{ij}={\left(K_{ij}\right)}^{r_{j}\cdot r_{i}}$. The value $K_{ij}$ is also the core to generate the response parameters. As described in Section 6.2.3, $K_{ij}$ is only accessible to $U_{i}$ and $S_{j}$—others could obtain it unless it compromises the secret key $X_{c}$ of RC. Therefore, server spoofing attack is meaningless in our scheme.

#### 6.2.5. Replay Attacks

The replay attack signifies that someone spitefully resubmits repeated or delayed messages to deceive honest participants for nefarious purposes. Timestamping is one of the most widely employed techniques to prevent replay attack. In our proposal, both login request and replied authentication messages are involved in current timestamp. Both participants can verify its validity by detecting message transmitting delay. As a consequence, the replay attack is resisted effectively.

#### 6.2.6. Forward Secrecy

Forward secrecy of information exchange protocol safeguards the past sessions to be revealed in which the long term key of RC is compromised in the future, even if the adversary actively interfered. Our proposed scheme achieves forward secrecy because the session key $SK_{ij}={\left(K_{ij}\right)}^{r_{j}\cdot r_{i}}$ is surrounded by $r_{i}$ and $r_{j}$. Even though the adversary calculates $K_{ij}$ with the leaked key $X_{c}$, he/she also is unable to further compromise $SK_{ij}$ computed with the contribution of one-time random numbers $\left\{r_{i},r_{j}\right\}$.

## 7. Performance and Functionality Analysis

Herein, we present performance and functionality evaluation analysis of our proposed scheme and other recently related protocols, that is, Chuang et al.’s scheme [16], Kumari and Om’s scheme [23] and Jangirala et al.’s scheme [26]. Table 2 and Table 3 show the comparative study in terms of security features and computational cost of the proposed scheme along with the aforementioned schemes, separately.

According to the comparisons of Table 2, we can see that our scheme satisfies all the requirements and criterion for multiple servers based authentication system. In contrast, the other three schemes suffer from more or less susceptibilities, even the superiorities claimed by the authors. In the modified scheme, we eliminate these flaws and enhance the security by targeted renovation.

In Table 3, the notations $T_{h}$ and $T_{e}$ denote the consuming time for a one-way hash function and a modular exponential operation, respectively. The evaluation shown in Table 3 focuses on the login phase, authentication phase and neglects the other three phases which do not frequently need to be performed. Chuang et al.’s and Jangirala et al.’s schemes use symmetric encryption and only perform hash function. Our proposed scheme and Kumari & Om’s scheme are employed by RSA cryptosystem and require to execute modular exponentiation. Thus, the latter two schemes need to expend more computational cost. From Table 3, the total computation cost of Chuang et al.’s scheme, Kumari & Om’s scheme, Jangirala et al.’s scheme and our scheme are 17$T_{h}$, $9T_{h}+7T_{e}$, 25$T_{h}$ and 8$T_{h}$ + 6$T_{e}$. Noticeably, our scheme can thwart many security threats identified on these schemes. Additionally, our scheme is proved formally with the BAN-logic.

## 8. Conclusions

This paper firstly identified that Kumari and Om’s anonymous multi-server authenticated key agreement scheme was plagued by impersonation attack, server spoofing attack and privacy disclosure. Even worse, any attacker could decipher it by launching a malicious attack without the knowledge of the victim’s secret information. Secondly, we introduce a modified multiple servers architecture based authentication scheme with biometric to rectify these security flaws. Subsequently, to evaluate the devised scheme, we present the formal proof validated by BAN-logic and logical analysis for a range of network attacks. The performance and functionality comparisons in terms of computational cost and security features show that the designed protocol is superior for multiple servers authentication system.

## Figures and Tables

**Table 1:** Notations.

$U_{i}$	User
$S_{j}$	Service providing server
RC	Registration center
$ID_{i}$	Identification of user
$PW_{i}$	Password of user
$SID_{j}$	Public identification of server
$B_{i}$	Biometrics information of user
$X_{c}$	Secret key of registration center
p,q	Two distinct large primes
n,ϕ(n)	n=p×q, ϕ(n)=(p−1)×(q−1)
$SK_{ij}$	Session key shared between user and server
H(·)	Hash function
⊕	Exclusive-OR operation
∥	String concatenation operation

**Table 2:** Comparisons of functionality.

	[16]	[23]	[26]	Ours
Prevention of impersonation attack	No	No	No	Yes
Prevention of off-line password guessing attack	Yes	No	Yes	Yes
Prevention of server spoofing attack	No	No	No	Yes
Preserving user privacy	Yes	No	No	Yes
Prevention of replay attack	No	Yes	Yes	Yes
Formal security proof	No	Yes	Yes	Yes
Mutual authentication	No	No	No	Yes
Smart card breach attack	Yes	No	No	Yes
Perfect forward secrecy	No	Yes	Yes	Yes

**Table 3:** Performance comparisons.

	[16]	[23]	[26]	Ours
Login phase	4$T_{h}$	$4T_{h}+2T_{e}$	8$T_{h}$	4$T_{h}$ + 2$T_{e}$
Authentication phase	13$T_{h}$	$5T_{h}+5T_{e}$	17$T_{h}$	4$T_{h}$ + 4$T_{e}$
Computation cost	17$T_{h}$	$9T_{h}+7T_{e}$	25$T_{h}$	8$T_{h}$ + 6$T_{e}$
